# Overexpression of a truncated form of the *MSN2 *gene enhances the initial rate of ethanol production in an industrial fuel-ethanol *Saccharomyces cerevisiae *strain

**DOI:** 10.1186/1753-6561-8-S4-P126

**Published:** 2014-10-01

**Authors:** Augusto Bücker, Davi Ludvig Gonçalves, Júlio Cézar Espírito Santo, Boris Stambuk

**Affiliations:** 1 Department of Biochemistry, Federal University of the Santa Catarina - UFSC, Florianópolis, SC, 88040-970, Brazil; 2 Instituto Tecnológico Vale - ITV, Belém, PA, 66055-090, Brazil

## Background

The yeast strain CAT-1 isolated from a Brazilian fuel-ethanol plant (Babrzadeh et al. 2009) is one of the most common strain used nowadays due to its very efficient fermentation capacity, especially at high sugar concentrations and under the stressful industrial conditions. Since the transcription factor genes *MSN4, MSN2, YAP1 *and *HSF1 *of tolerant yeast strains are highly expressed under ethanol stress [1], we generated a CAT-1 derived strain named ATT-6 that overexpresses a truncated form of the transcription activator Msn2 through genomic engineering and analyzed the ethanol stress tolerance and fermentation capacity of this modified strain.

## Methods

Following the procedure described by Petracek and Longtine [2], for the construction of a yeast strains overexpressing a truncated form of the *MSN2 *gene, a DNA fragment containing the *Kan*^r ^which confers resistance to G418 (geneticin), flanked by *LoxP *regions and the constitutive PADH1 promoter, was integrated into the genomic locus of the *MSN2 *gene of CAT-1, deleting the N-terminal region (first 48 amino acids) of the protein. The effect of 12-16% (v/v) ethanol addition on cell growth of the strain was evaluated in 96-well plates using a Tecan GENios microplate reader at 30°C and 110 rpm. Fermentation performance was determined using high sucrose concentration (200 g/L).

## Results and conclusions

Under microaerobic conditions, the industrial ATT-6 strain overexpressing a truncated *MSN2 *gene showed increased growth rates and increased tolerance to up to 16% (v/v) ethanol stress, while the industrial control CAT-1 strain did not show cell growth before 60 hours. These results were consistent with those obtained by Hong et al. [3], indicating that overexpression of a truncated *MSN2 *can increase ethanol tolerance in a laboratory *S. cerevisiae *strain. Next, we investigated whether this improvement in cellular viability under high ethanol conditions leads to higher ethanol productivities during ethanol fermentation. We used high sucrose concentration (200 g/L) to expose yeast cells to high ethanol concentrations, caused not only by the initially added ethanol (12-16%), but also by the ethanol produced during fermentation. Our results show that the genetically modified strain ATT-6 grew slightly better than the control strain in the presence of supplemented ethanol. Ethanol production of the ATT-6 strain also showed slight initial higher ethanol productivity during the first 8 hours, when compared to the parental strain. Furthermore, the ATT-6 strain also presents higher invertase activity needed for the sucrose consumption, a result in accordance with Geng and Laurent [4] that indicates that Msn2/Msn4 act specifically in the early phase of *SUC2 *induction. Since there is limited information available for the function of the truncated form of *MSN2*, further studies on its regulatory roles for ethanol tolerance are needed. In conclusion, our results show that the industrial ATT-6 strain that overexpresses the truncated *MSN2 *allele is more tolerant to ethanol and produced more ethanol than the control unmodified strain.

## References

[B1] Ma M Liu ZL Quantitative transcription dynamic analysis reveals candidate genes and key regulators for ethanol tolerance in Saccharomyces cerevisiae BMC Microbiol 2010 10 169 10.1186/1471-2180-10-169 20537179 PMC2903563

[B2] Petracek ME Longtine MS PCR-Based Engineering of Yeast Genome Methods Enzymol 2002 35 445 469 12073329 10.1016/s0076-6879(02)50978-2

[B3] Hong M Lee K Yu BJ Sung Y Park SM Koo HM Identification of gene targets eliciting improved alcohol tolerance in saccharomyces cerevisiae through inverse metabolic engineering J Biotechnol 2010 149 52 9 10.1016/j.jbiotec.2010.06.006 20600383

[B4] Geng F Laurent BC Roles of SWI/SNF and HATs throughout the dynamic transcription of a yeast glucose-repressible gene EMBO J 2004 23 127 37 10.1038/sj.emboj.7600035 14685262 PMC1271673

[B5] Babrzadeh F Jalili R Wang C Shokralla S Pierce S Robinson-Mosher A Nyren P Shafer RW Basso LC Amorim HV Oliveira AJ Davis RW Ronaghi M Gharizadeh B Stambuk BU Whole-genome sequencing of the efficient industrial fuel-ethanol fermentative Saccharomyces cerevisiae strain CAT-1 Mol Genet Genomics 2012 287 485 494 22562254 10.1007/s00438-012-0695-7

